# Cocaine Anæsthesia

**Published:** 1889-07

**Authors:** John L. Gish

**Affiliations:** Jackson, Mich.


### Cocaine Anæsthesia.—With a Report of over Fifty Cases.

JOHN L. GISH, M.D., D.D.S., JACKSON, MICH.

Read before the Michigan State Dental Society, January 5th, 1889.

I am prompted to present this article by the many adverse criticisms on cocaine anæsthesia, that have appeared in the medical and dental journals in this country and on the continent. They have portrayed the danger of its use in the most graphic manner and have given to it but a small share of credit that is its due so far as anæsthesia is concerned.

It has not been without profit that our attention has been called to its poisonous effects, and unpleasant symptoms as a result of its administration, but at the same time the danger signal has been hoisted too many times without giving a more rational explanation of its cause.

Cocaine is a drug that is to be used with some degree of *caution*, and to be *administered understandingly*; but this same fact applies to *all* potent drugs found in both the mineral and vegetable worlds. Cocaine in the form of wine of cocoa is recognized and prescribed by the medical world as a most valuable nerve tonic, and as an aid to convalescing patients. The ophthalmologist uses cocaine with the most brilliant results, and yet from that specialty you will occasionally see reported a bad case of acute sore eye from the effect of cocaine applications without any other apparent cause. Whereas, if the true cause had been found it would not have been cocaine, as cocaine, but a decomposed solution that had been prepared many hours and perhaps weeks. Now, without going into the history, pysiology, and toxicology of the drug I will simply give my experience of its use as administered to meet the wants of the oral and dental surgeon.

Anxious to meet the wants of suffering humanity and alleviate pain in such operations as the oral and dental surgeon is frequently called upon to perform, I hail with much joy the first flattering reports that teemed the journals and welcomed the new drug to a place in my cabinet some four years ago.

If I may be permitted to use the expression, I must say that I was "in the swim." I could see in cocaine a great boon to the afflicted as well as to the dental surgeon. Stimulated by the brilliant results of the ophthalmologist, whose work is upon the delicate structures of the eye, I could see cocaine act upon the more dense structures of the mouth in the same manner. And so the first few cases did happen to be so, and of a natural consequence the problem was solved in my mind. But by and by the first stage of excitement passed away and then I had similar results to my confreres in my part of the country: namely, now and then a good result, but more often a failure in part or whole, with occasional unpleasant symptoms of nausea, vertigo, and general numbness with more or less anxiety.

The good results that I did have were just enough to stimulate me to its farther use, and a more thorough understanding of the nature of the drug, and a more rational explanation of its seemingly bad results. The first series of experiments led me to the conclusion that the unpleasant symptoms were due to an imperfect drug. This opinion I held for some time; until I knew that I was using a good article, which by the way, is even now difficult to obtain inasmuch as it is adulterated with bicarbonate of soda. The unpleasant symptoms would occasionally present themselves even with a known good article. Suggestion after suggestion would come to my mind and remain until its fallacy was proven.

I studied the temperaments of both sexes with the idea that I might so modify the dosage that I could overcome and bring about the desired result; and here I observed with profit that the blond type, speaking in a general way, were the most susceptible to the drug and the more often gave unpleasant symptoms. But yet the end was not complete. Hence with the facts already observed I began to study the *time of administering cocaine relatively to the meal hour* of the patient and found that I had no unpleasant symptoms when the patient came to the office shortly after eating his or her breakfast or dinner, but *did* have occasionally the bad results if they came before their dinner or supper. With this fact before me I was led to observe a little further: namely, as to whether the patient to whom I was about to administer cocaine had or had not stopped on his or her way and imbibed a little something, just to help them up the stairs. From this observation I learned that he who had imbibed had no bad results even though it was just before his meal hour.

With these observations I have decided that in order to have success with cocaine anæsthesia, as desired by the oral and dental surgeon, he must study the temperament of his patient; he must know the quality and strength of his drug; he must consider the hour of administration, and if it is more than an hour after the patient has partaken of food, before using any cocaine the patient should be given from two to four fluid drachms of spiriti vini gallici.

Before giving the mode of administration I will give the rational explanation, as it occurs to me, why we should observe the above precautions in order to have success.

1st. According to clinical teaching we must vary our dosage with the age and temperament of our patient.

2nd. With the purity and potency of our drug do we get the required effect.

3rd. Nausea and vertigo with the feeling of numbness being caused by a peripheral irritation of the pneumogastric nerve when the stomach is not engaged in the process of digestion, then the brandy or any of its substitutes will prevent this irritation, which in turn will relieve our patients of the above unpleasant symptoms. (We must not confound the general signs of fainting with the nausea, vertigo, etc., caused by cocaine, nor must we forget the influence of mind over body).

Mode of Administration.—I always use a freshly prepared solution of muriate of cocaine. From a three to a ten per cent. solution, or stronger if the patient requires it. With a little pledget of cotton I bathe the mucous membrane over the parts to be operated on. After an interval of about two minutes I inject, without pain, by means of a very small hypodermic syringe from one to two drops beneath the mucous membrane. After an interval of two or three minutes I inject one or two drops more, down deeper into the tissues. The injecting is repeated two or three times. After a lapse of ten or fifteen minutes, sometimes twenty minutes from the time you used the cocaine with the pledget of cotton the parts will be thoroughly anæsthetized. Of course, the susceptibility of the patient, will govern the time before you can operate.

With these few facts in my possession, I will submit them with a record of over fifty cases, to the scientific men of our profession and await their decision as to whether I have been successful or not.

Record of cases as taken from my book of records under the division of Cocaine Anæsthesia.

Case 1. Mrs. Q. city, ulcerated central and lateral incisor roots removed at one sitting without the least bit of pain. Cocaine ten per cent. solution applied locally and hypodermically. Time, twenty minutes. No bad symptoms.

Case 2. Mrs. R. Buffalo, N. Y., roots of the left upper wisdom tooth badly ulcerated and very sore; removed by means of the elevator; extracting by aid of the forceps being impossible. Very little pain. Cocaine ten per cent. solution locally and hypodermically applied; time twenty-five minutes.

Case 3. Mr. G. S. B. city, root of right upper first bicuspid removed by aid of surgical means; very little pain at first but much exhaustion from prolonged operation. Cocaine and ether spray; time one hour.

Case 4. Mr. W. F. McG. Root of supernumary left lower bicuspid removed by surgical means alone; very difficult; no pain to speak of. Cocaine ten per cent. solution locally and hypodermically applied; time one hour; no bad symptoms.

CASE 5. Master E. P. Left lower first molar removed without any pain. Cocaine ten per cent. solution locally and hypodermically applied; time eighteen minutes.

CASE 6. Master N. W. Removed lower left first molar without pain. Cocaine ten per cent. solution locally and hypodermically applied; time twenty minutes.

CASE 7. Mrs. M. A. D. Removed left lower wisdom tooth, badly ulcerated, without pain; time fifteen minutes. Cocaine ten per cent. solution locally and hypodermically applied.

CASE 8. Mrs. H. S. Removed four ulcerated roots at one sitting without pain. Cocaine ten per cent. solution locally and hypodermically applied; time thirty minutes.

CASE 9. Mrs. D. S. Removed diseased incurable right lower second molar tooth; no pain whatever; time twenty minutes. Cocaine ten per cent. solution applied locally and hypodermically. No unpleasant results.

CASE 10. Miss E. B. Removed left lower first molar in an incurable condition; caused by the mercury in a large silver filling; time twenty-five minutes. Cocaine ten per cent. solution hypodermically applied; no pain.

CASE 11. Mrs. M. A. B. Removed left lower wisdom tooth badly decayed and ulcerated; no pain whatever. Cocaine ten per cent. solution hypodermically applied; time twenty minutes.

CASE 12. Miss E. B. Removed right upper first molar in an incurable condition; caused by the mercury in a large silver filling; time twenty minutes. Cocaine hypodermically applied; no pain whatever.

CASE 13. Mrs. B. S. M. Removed right and left lower first molar complicated with cystic tumors. The cyst and tooth of the left side were of a much more grave character than on the right. The diseased conditions were caused by mercurial poisoning from large silver fillings that these teeth contained. Cocaine applied locally and hypodermically; used freely; no bad results and no pain whatever.

CASE 14. Mr. T. J. B. Removed left lower second molar. Incurable. Cocaine hypodermically applied; no pain; time twenty minutes.

CASE 15. Mr. F. B. Removed right lower second molar badly ulcerated and painful even to touch of tongue. No pain; time twenty minutes. Cocaine locally and hypodermically applied.

CASE 16. Mrs. R. S. S. Removed two roots that were decayed below surface of gum; no pain; time thirty minutes. Cocaine locally and hypodermically applied.

CASE 17. Mrs. G. M. Removed one ulcerated root that was decayed far below surface of gum; no pain; time twenty minutes. Cocaine applied locally and hypodermically.

CASE 18. Mr. A. W. Removed right upper wisdom tooth badly ulcerated; very sore to the touch; no pain; time twenty minutes. Cocaine ten per cent. solution applied locally and hypodermically.

CASE 19. Master C. W. Removed four premolars at diffierent times. Badly ulcerated and interlocked with the crowns of the permanent teeth below them, making extraction very long, tedious, and difficult; no pain; time thirty minutes for each tooth. Cocaine ten per cent. solution applied locally and hypodermically.

CASE 20. Mr. C. H. Removed right lower first molar, ulcerated and crown thoroughly honeycombed with dental caries. No pain; time twenty minutes. Cocaine ten per cent. solution locally and hypodermically applied.

CASE 21. Mrs. A. N. H. Removed ulcerated roots of first and second left upper bicuspids; no pain; time twenty-five minutes. Cocaine ten per cent. solution hypodermically applied.

CASE 22. Mrs. M. McG. Removed ulcerated root of left lower first bicuspid; root decayed beneath surface of gum; no pain; time twenty minutes. Cocaine ten per cent. solution applied locally and hypodermically.

CASE 23. Miss M. H. Removed anterior root of left upper first premolar, engaged on the crown of the permanent tooth beneath, or rather beside it in this case; no pain; time eighteen minutes. Cocaine ten per cent. solution applied locally and hypodermically.

CASE 24. Miss M. H. Removed right upper first molar, ulcerated; very sore; no pain; time twenty minutes. Cocaine ten per cent. solution locally and hypodermically applied.

Case 25. Mrs. J. M. S. Removed ulcerated roots of upper central incisors, left upper lateral incisor, and right and left upper second molars at one sitting; no pain whatever until the left upper second molar, which was removed last, and then the pain that was felt was very slight; time forty-five minutes. Cocaine, ten per cent. applied locally and hypodermically.

Case 26. Miss K. W. Removed badly decayed left upper first bicuspid on account of irregularity; no pain; time twenty-five minutes. Cocaine ten per cent. solution applied locally and hypodermically.

Case 27. Mrs. C. K. L. Removed the diseased roots of left lower central and lateral incisor, and roots of right lower first and second bicuspid, and second molar; also a malformed wisdom tooth by surgical means at one sitting; no pain whatever except a very little in the removal of the right lower lateral, caused by commencing the operation before complete anæsthesia. Time one hour and fifteen minutes.

Case 28. Miss M. H. Removed left lower first molar badly decayed, for irregularity; no pain; time twenty minutes. Cocaine ten per cent. applied locally and hypodermically.

Case 29. Mr. S. E. Removed right lower first molar badly ulcerated; no pain; time twenty minutes. Cocaine ten per cent. solution applied hypodermically.

Case 30. Mr. D. S. Removed left upper wisdom tooth badly ulcerated; no pain to speak of; parts not completely anæsthetized; time twenty minutes. Cocaine applied locally and hypodermically.

Case 31. Mrs. W. H. McA. Removed ulcerated roots of left upper first and second bicuspids; no pain; time twenty-five minutes. Cocaine ten per cent. solution applied locally and hypodermically.

Case 32. Mrs. M. G. C. Removed the four lower incisors; roots recrossed and abraded; no pain; time twenty-five minutes. Cocaine ten per cent. solution locally and hypodermically applied.

Case 33. Miss A. DeL. Removed left upper second bicuspid for irregularity; no pain; time thirty minutes. Cocaine ten per cent. solution locally and hypodermically applied.

Case 34. Master C. G. Removed left upper first molar; ulcerated and badly decayed; no pain; time twenty minutes. Cocaine locally and hypodermically applied.

Case 35. Miss H. M. P. Removed right lower first molar; ulcerated; very sore; no pain; time twenty minutes. Cocaine ten per cent. solution applied locally and hypodermically.

Case 36. Mr. J. E. Removed left lower first molar; ulcerated; very difficult; no pain; time twenty-five minutes. Cocaine ten per cent. solution applied locally and hypodermically.

Case 37. Mrs. H. W. S. Removed ulcerated roots of left upper first and second bicuspids; no pain; time twenty-five minutes. Cocaine ten per cent. solution applied locally and hypodermically.

Case 38. Miss B. T. Removed nine temporary teeth on account of nervous irritation. Three of the above teeth were ulcerating. The teeth were removed in six appointments; no pain except in the removal of one cuspid which was very firmly imbedded and not completely anæsthetized. The average time for each sitting was about twenty minutes. Cocaine ten per cent. solution applied locally and hypodermically except in the case that caused pain; then it was used only locally.

Case 39. Mr. D. S. Removed left upper first molar; badly ulcerated; no pain; time twenty minutes. Cocaine applied locally and hypodermically.

Case 40. Mr. J. G. Removed right lower wisdom tooth; badly ulcerated; no pain; time twenty minutes. Cocaine ten per cent. solution applied locally and hypodermically.

Case 41. Mr. E. J. C. Removed right lower wisdom tooth; badly ulcerated; no pain; time twenty minutes. Cocaine ten per cent. solution applied locally and hypodermically.

Case 42. Mr. A. B. T. Removed the roots of the right and left upper cuspids, right and left upper laterals, and right and left upper central incisors, and the roots of the left upper first molar at one sitting. The roots all badly diseased and firmly adhered to the surrounding tissues by chronic inflammatory adhesion; no pain; time one hour. Cocaine ten per cent. solution applied locally and hypodermically.

CASE 43. On the following day I removed ten roots from the lower jaw of the same patient. The character of the roots the same as those removed on the previous day from the upper maxillary, and the result was the same with the exception of a very little pain when the badly ulcerated roots of the right and left first bicuspids were removed by means of the elevator and chisel.

CASE 44. Mrs. M. G. Removed right lower wisdom tooth; ulcerated and necrosed roots; no pain; time twenty minutes. Cocaine applied locally and hypodermically.

CASE 45. Miss L. G. Removed two premolars in the first stages of ulceration, and roots engaged on the crown of the permanent teeth beneath; no pain; time thirty minutes. Cocaine ten per cent. solution applied locally and hypodermically.

CASE 46. Miss M. C. Removed right lower first molar; badly ulcerated; very sore and firmly fixed by chronic inflammatory tissue; no pain; time thirty minutes. The work was accomplished by means of the elevator and chisel. Cocaine ten per cent. solution locally and hypodermically applied.

CASE 47. Mr. W. Removed right lower first molar; difficult extraction; no pain; time twenty-five minutes. Cocaine ten per cent solution freely used locally and hypodermically.

CASE 48. Mr. S. C.; aet. 17. Removed second premolar of right lower jaw. The roots engaging the crown of the permanent teeth beneath it so as to make its removal very difficult; no pain; time twenty minutes. Cocaine applied freely locally and hypodermically.

CASE 49. Mr. C. R. K. Removed right upper wisdom tooth; ulcerated; no pain; time twenty-five minutes. Cocaine applied locally and hypodermically.

CASE 50. Miss C. C. Removed roots of left lower first molar by means of elevator and chisel. The roots firmly adhered and united to the surrounding tissues by chronic imflammatory adhesions of over a year's standing. There was a constant secreing and discharge of pus from the diseased structures. Time twenty minutes. Cocaine ten per cent. solution; ten gtts. of which three or four were used locally and the rest injected into the diseased structure. No pain whatever with the exception of a very little upon the removal of the anterior root which was largely exostosed and which caused a spreading of the alveolar process to the adjoining permanent teeth.

The above cases cover a period of several months but I think that it shows that when the most conservative of us in the art and science of saving the dental organs are called upon to perform such operations as the above, we have at our command an agent by the aid of which we can mitigate pain without danger if properly handled.

---

DISCUSSION.

Dr. J. Taft opened the discussion and said that the reports as read by Dr. Gish were very encouraging, to the use of the drug. The experiments were more practical and satisfactory, not being performed on the lower animals. Great care ought to be exercised in the use of the drug even when used on the surface locally. It should be procured from a reliable source, and prepared carefully when wanted for use insolution. Upon inquiring as to the reason for waiting twenty or thirty minutes before operating, Dr. Gish replied that it took that length of time for the cocaine to affect the tissues and that the time depended upon the patient.

Dr. Harroun described a method he had been using, for the removal of the pulp with cocaine. He exposed as much of the pulp as possible, then applied the cocaine crystals, saturated with water, to the exposure. In ten cases there was no pain. He confined his attempts to anterior and bicuspid teeth.

The question was asked, What causes the sickness when cocaine is used in the extraction of teeth?

Dr. Gish thought it would be due to the idiosyncrasy of the patient as he had noticed the same sickness when nothing had been used to obtund the pain.

Dr. J. Taft related a case where a man, strong and healthy, had come to him to have a tooth extracted; while he was selecting an instrument the patient became deathly sick. Dr. Taft thought the method adopted by Dr. Harroun for the extirpation of the pulp should do away with the use of arsenious acid, which had been the cause of a great deal of mischief. Dr. Harroun said he did not promise the patient perfect freedom from pain ; simply told them it would mitigate it and help them along, and that the operation could often be completed at the same sitting.

Dr. Talbot, of Chicago, was called upon by the chair to take part in the discussion. Dr. Talbot spoke kindly of the reception given him by the Michigan Dental Association nine years ago at the twenty-fifth anniversary of the society. He had not used cocaine very much—did not have a great deal of faith in it. His practice did not permit of his waiting fifteen or twenty minutes for the drug to take effect; so when the patient wished cocaine he applied it, and used the forceps immediately with very good results.

